# Supplementary figures and images for: Modulation of the dynamics of cerebellar Purkinje cells through the interaction of excitatory and inhibitory feedforward pathways
Source: PLoS Comput Biol. 2021 Feb 10;17(2):e1008670. doi: 10.1371/journal.pcbi.1008670 (PMC7909957; doi:10.1371/journal.pcbi.1008670)

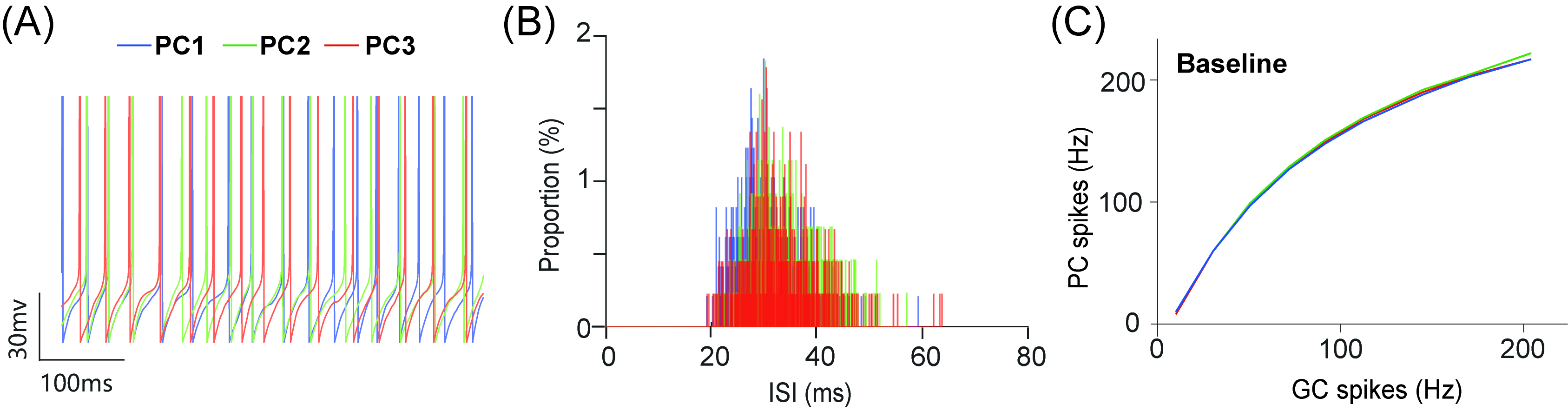

Supplement: S1 Fig — (A) Membrane potential traces of three example PCs triggered by 20 Hz Poisson spikes. (B) The corresponding ISI (interspike interval) distribution. (C) The input-output relationship represented by PC firing a function of input GC spikes. Here the simulation is conducted in the baseline condition, where there is no inhibitory MLI (MLI off) and no excitatory STP on GC-PC synapses (STP off, i.e., synaptic dynamics is not subjected to the modulation of short term plasticity). (TIF) [file pcbi.1008670.s001.tif]

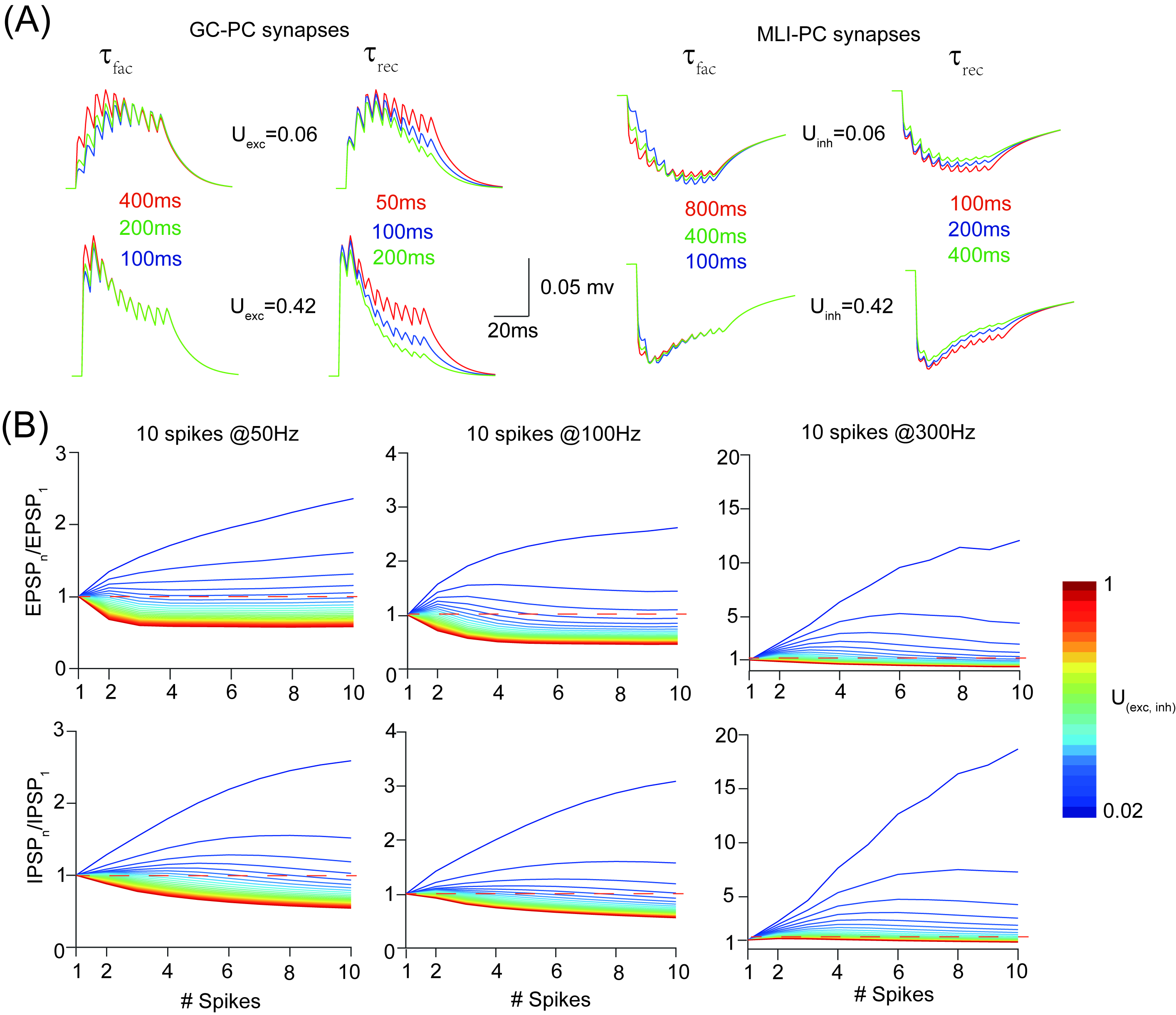

Supplement: S2 Fig — (A) The STP dynamics with different parameters of time constants: τfac for facilitation and τrec for depression, under two settings of initial efficacy U for GC-PC synapses and MLI-PI synapses. (Left) EPSPs triggered by a train of 10 spike at 200 Hz at Uexc = 0.06 for facilitation and Uexc = 0.42 for depression, with different facilitation time constants (τfac) and recovery time constants (τrec). (Right) Similar to EPSPs but for IPSPs with Uinh. (B) STP described by the ratio PSPn/PSP1 (EPSPs, top; IPSPs, bottom) showing facilitation or depression triggered by a train of different burst spikes at 50, 100 and 300 Hz with a wide range of U values (0.02-1). (TIF) [file pcbi.1008670.s002.tif]

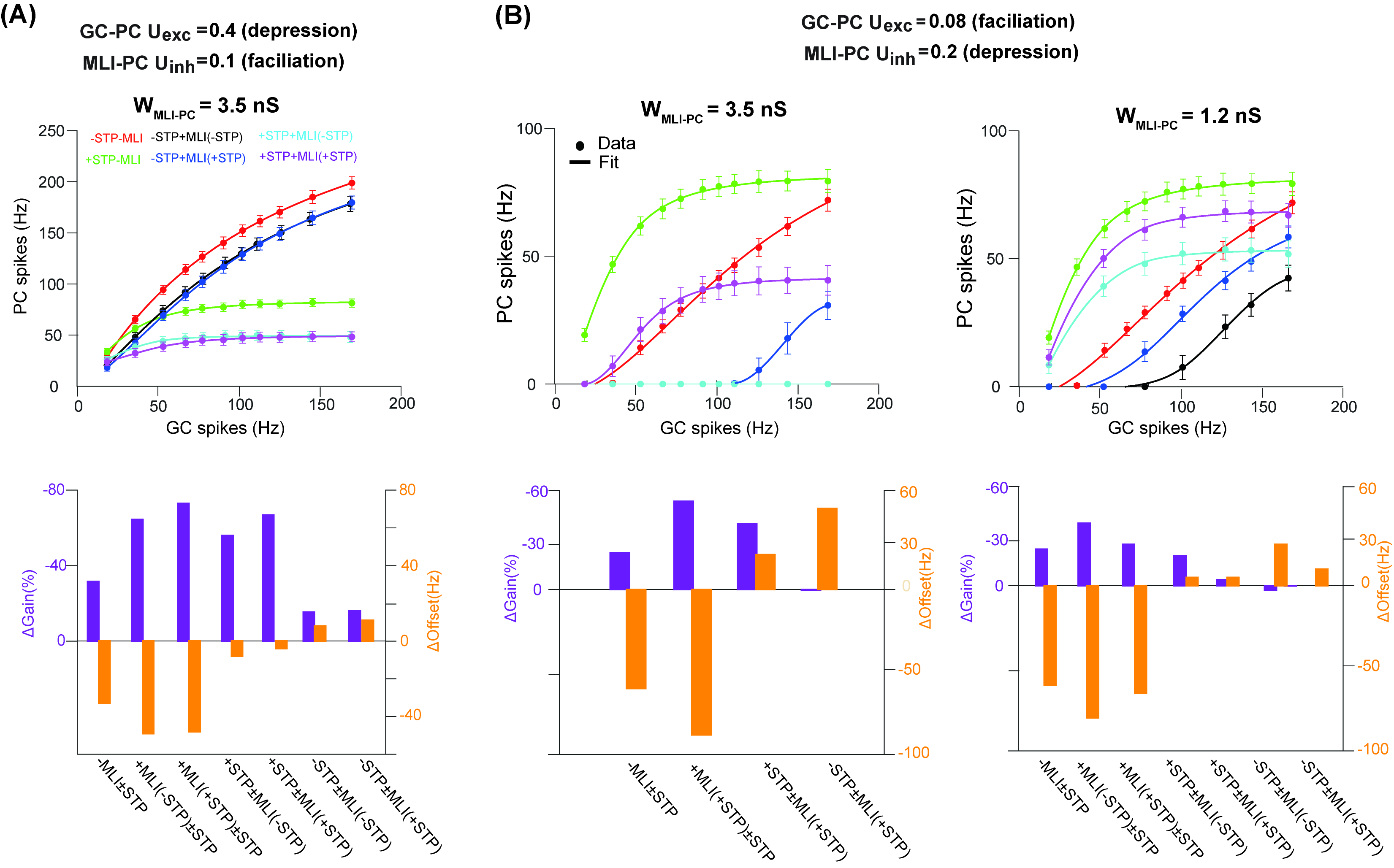

Supplement: S3 Fig — (A) The profiles of PC firing with an opposite (in contrast to the default values) pair of STP initial efficacy U values: Uexc = 0.08 for GC-PC facilitation and Uexc = 0.02 for MLI-PC depression, under different conditions. Here STP refers to GC-PC STP, and MLI refers to MLI-PC inhibition. Baseline without STP and MLI (-STP-MLI); STP ON without MLI (+STP-MLI); STP off with MLI but no MLI-PC STP (-STP+MLI(-STP)); STP off with MLI and MLI-PC STP (-STP+MLI(+STP)); STP on with MLI but no MLI-PC STP (+STP+MLI(-STP)); STP on with MLI and MLI-PC STP (+STP+MLI(+STP)). Each point is mean±SD (n = 50). (B) Similar to A but GC-PC synapses are depressing and MLI-PC synapses are facilitating. Here the STP of MLI-PC synapses is switched off, compared to Fig 2. WMLI-PC = 3.5 nS gives strong inhibition and depresses PC firing (left). The I-O profiles are more visible with WMLI-PC = 1.2 nS. Lines are fits to a Hill function. (TIF) [file pcbi.1008670.s003.tif]

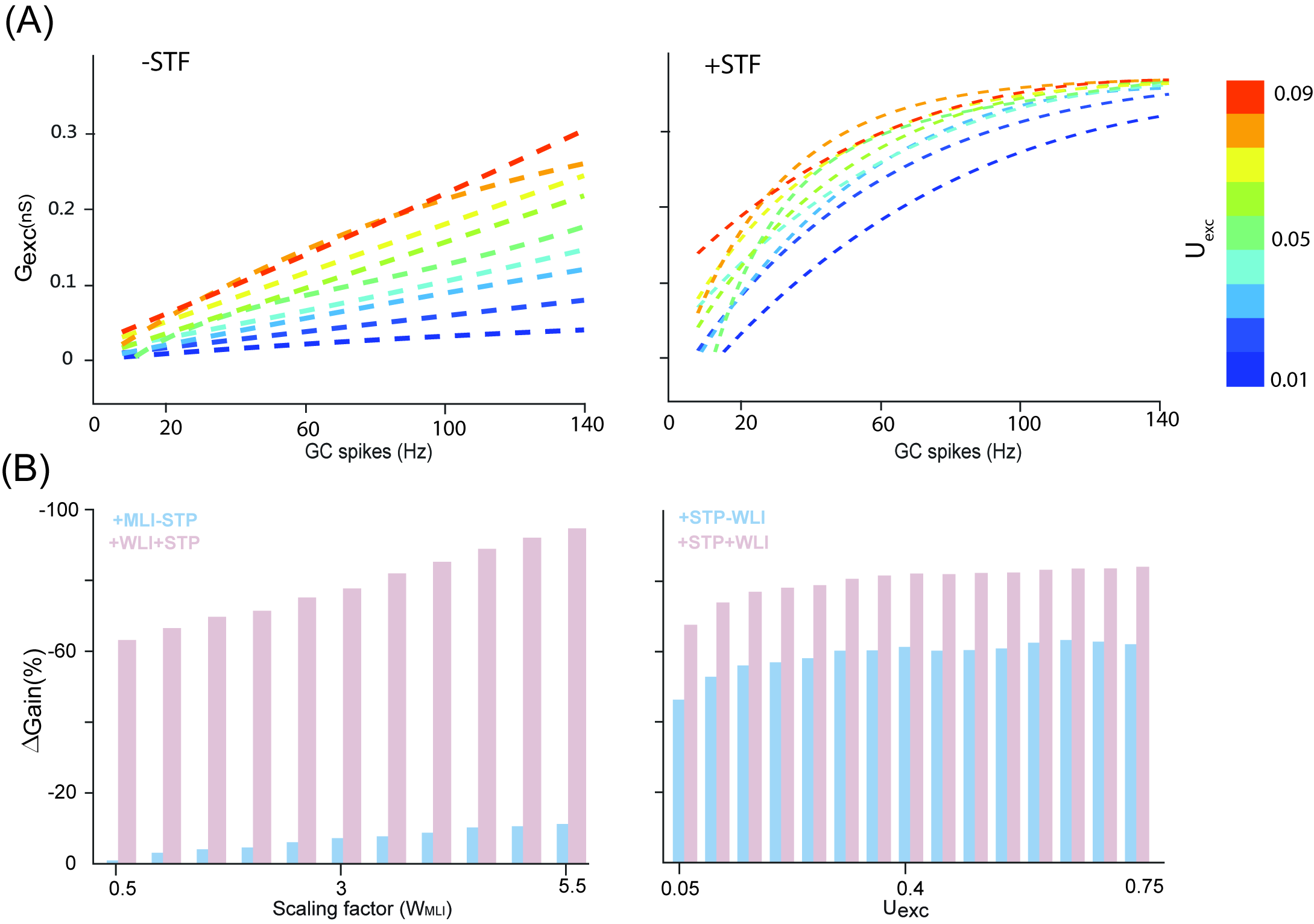

Supplement: S4 Fig — (A) The total excitation input Gexc as a function of GC input at various levels of synaptic efficacy Uexc (0.01-0.09) without short-term facilitation (STF) (left) and with STF (right). (B) The gain change without and with STP for different levels of inhibition (left), and without and with inhibition for different levels of STP Uexc (right), compared to the baseline for each case. The MLI inhibition weights are changed by a scaling factor as shown in the axis index. The default parameter values are used here, such that +STP means Uexc = 0.4, and +MLI means WMLI = 3.5 nS. (TIF) [file pcbi.1008670.s004.tif]

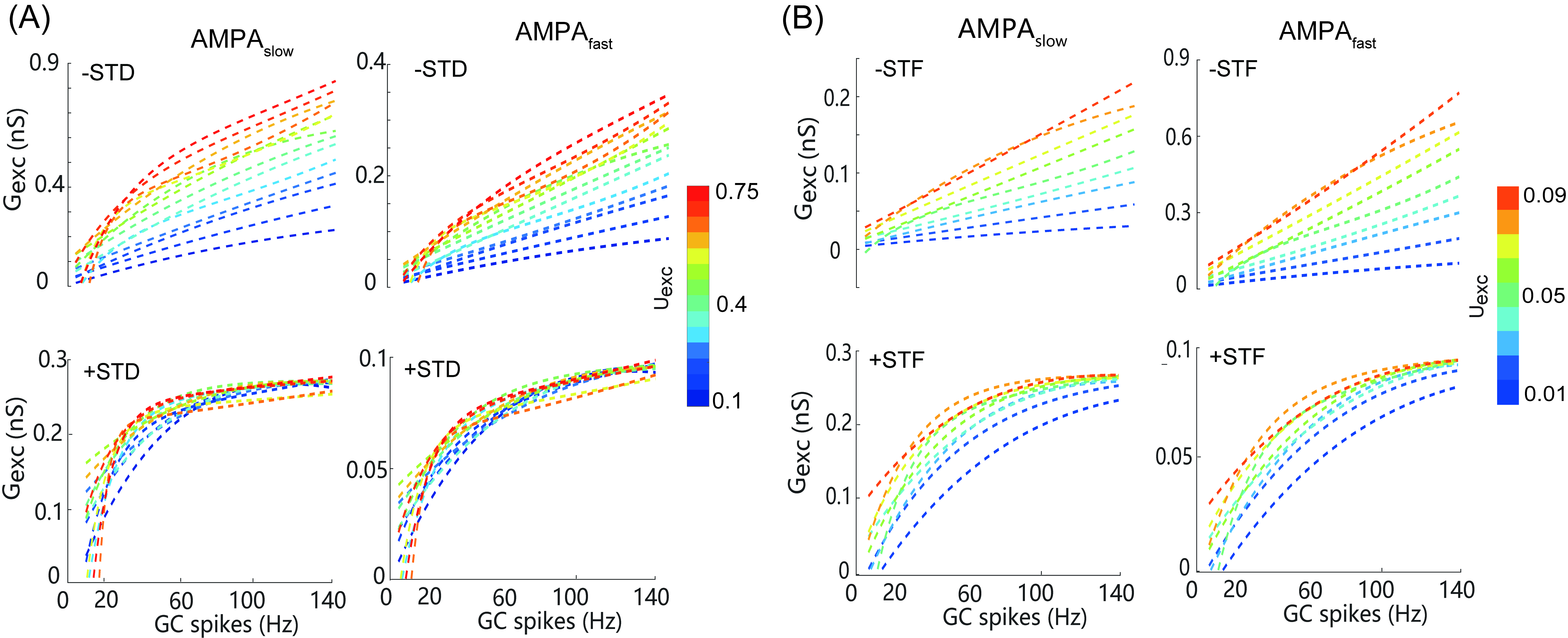

Supplement: S5 Fig — (A) Excitation Gexc of AMPA slow component (left) and AMPA fast component (right) as a function of GC input at various levels of synaptic efficacy Uexc (0.1-0.75) without short-term depression (STD) (top) and with STD (bottom). (B) Similar to (A) but for lower values of Uexc (0.01-0.09) without short-term facilitation (STF) (top) and with STF (bottom). (TIF) [file pcbi.1008670.s005.tif]

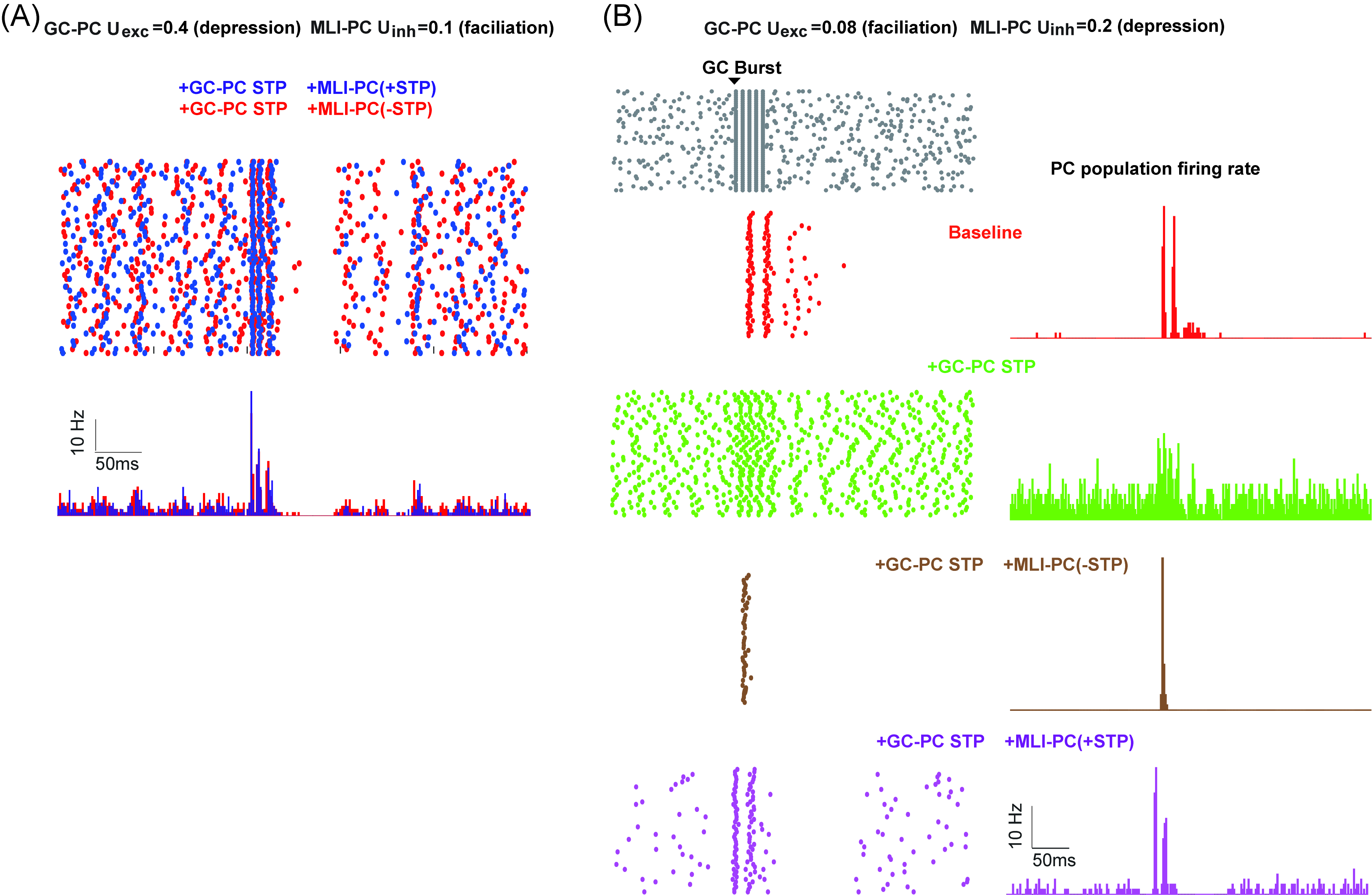

Supplement: S6 Fig — (A) The PC network shows similar behaviors with (blue) and without (red) MLI-PC STP. (B) Similar to Fig 7, but with an opposite pair of U values: Uexc = 0.08 for GC-PC facilitation and Uexc = 0.2 for MLI-PC depression, in contrast to Fig 7. Without short-term depression in MLI-PC synapses, the inhibition is too strong to suppress PC firing, which indicates the need of a balance of excitation and inhibiting, as also revealed by Figs 9–11. (TIF) [file pcbi.1008670.s006.tif]

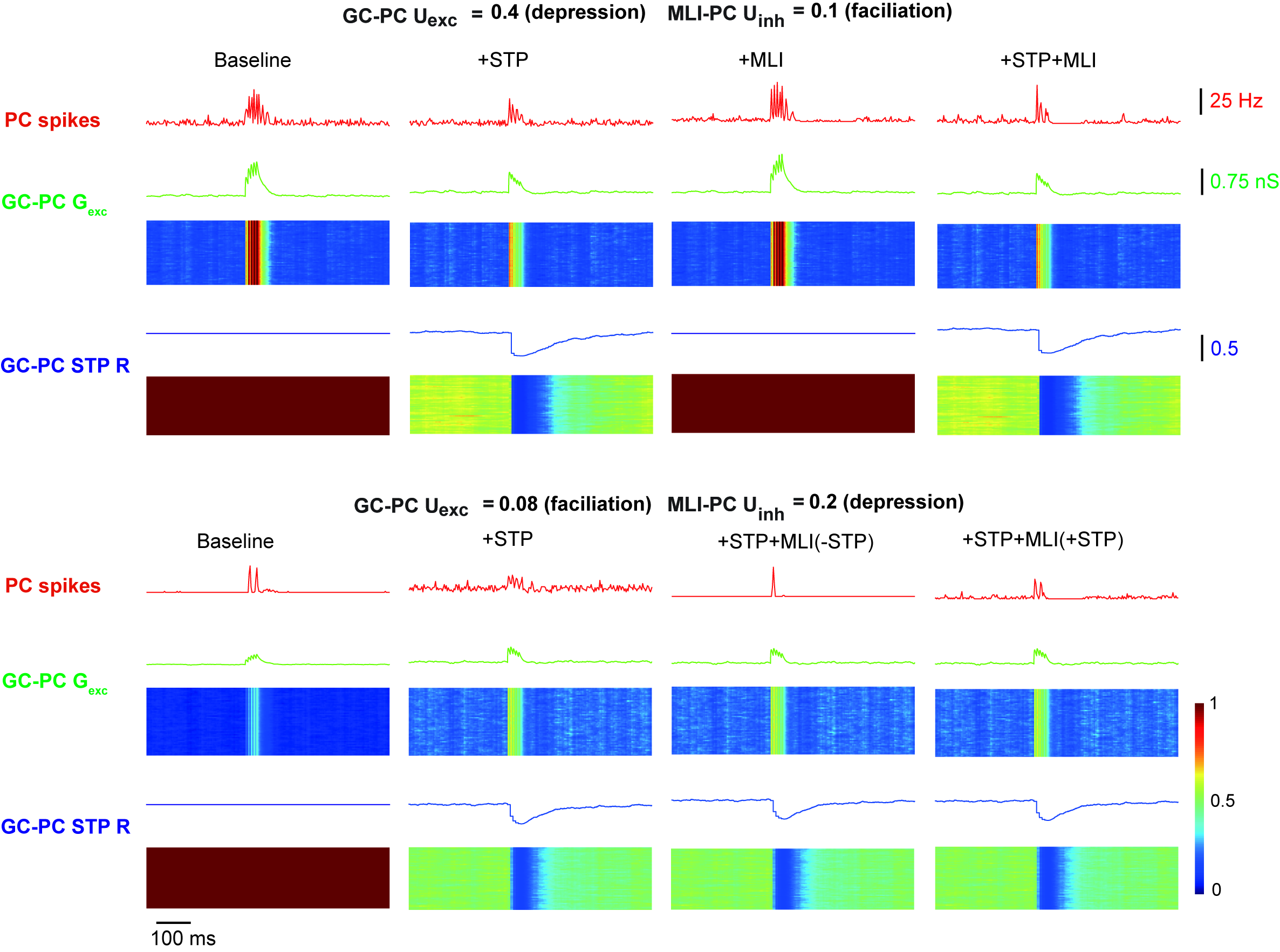

Supplement: S7 Fig — Time courses of PC population firing rate (red), total excitatory conductance averaged over all PCs (Gexc, green) and raster plots of excitatory conductance of each PC, and short-term plasticity R variable averaged over all PCs (blue) and raster plots of R of each PC. The same pairs of U values under different settings as in S6 Fig GC-PC synapses are depressed due to STP, and R variable reduced to be close to 0 during burst inputs. (TIF) [file pcbi.1008670.s007.tif]

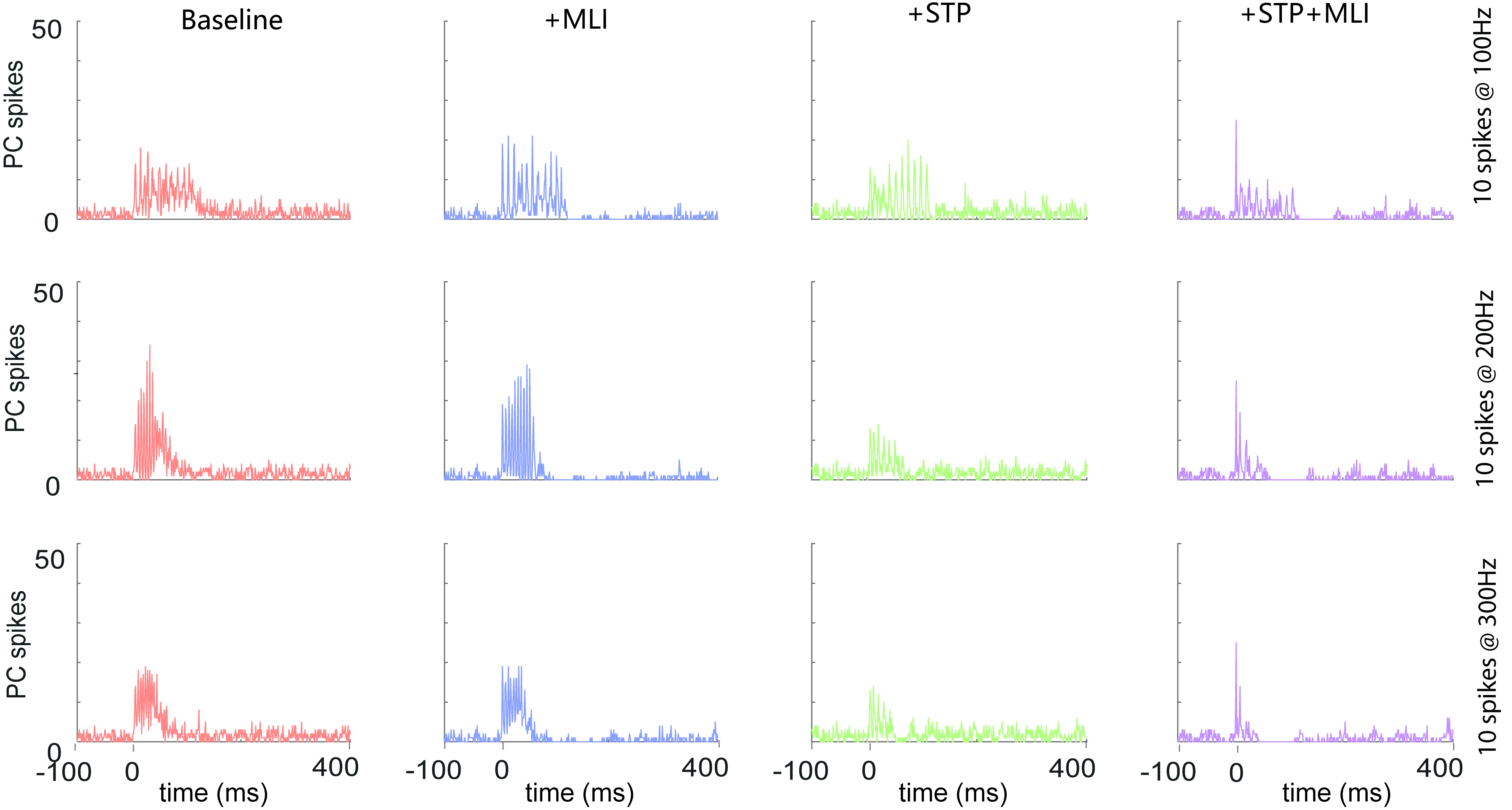

Supplement: S8 Fig — PC population firing rate under bursts of 10 spikes at 100, 200, and 300 Hz in four different conditions, baseline, with MLI, with STP, and with both MLI and STP. The background Poisson stimulation frequency is 20 Hz. The PC population firing rate tends to be sustained in the baseline. Adding MLI tends to build up dynamics over stimulus, in particular for high frequencies. Adding STP tends to make dynamics transient and decay over stimulus. Using MLI and STP together makes the dynamics more transient, so that the first peak is more prominent. (TIF) [file pcbi.1008670.s008.tif]

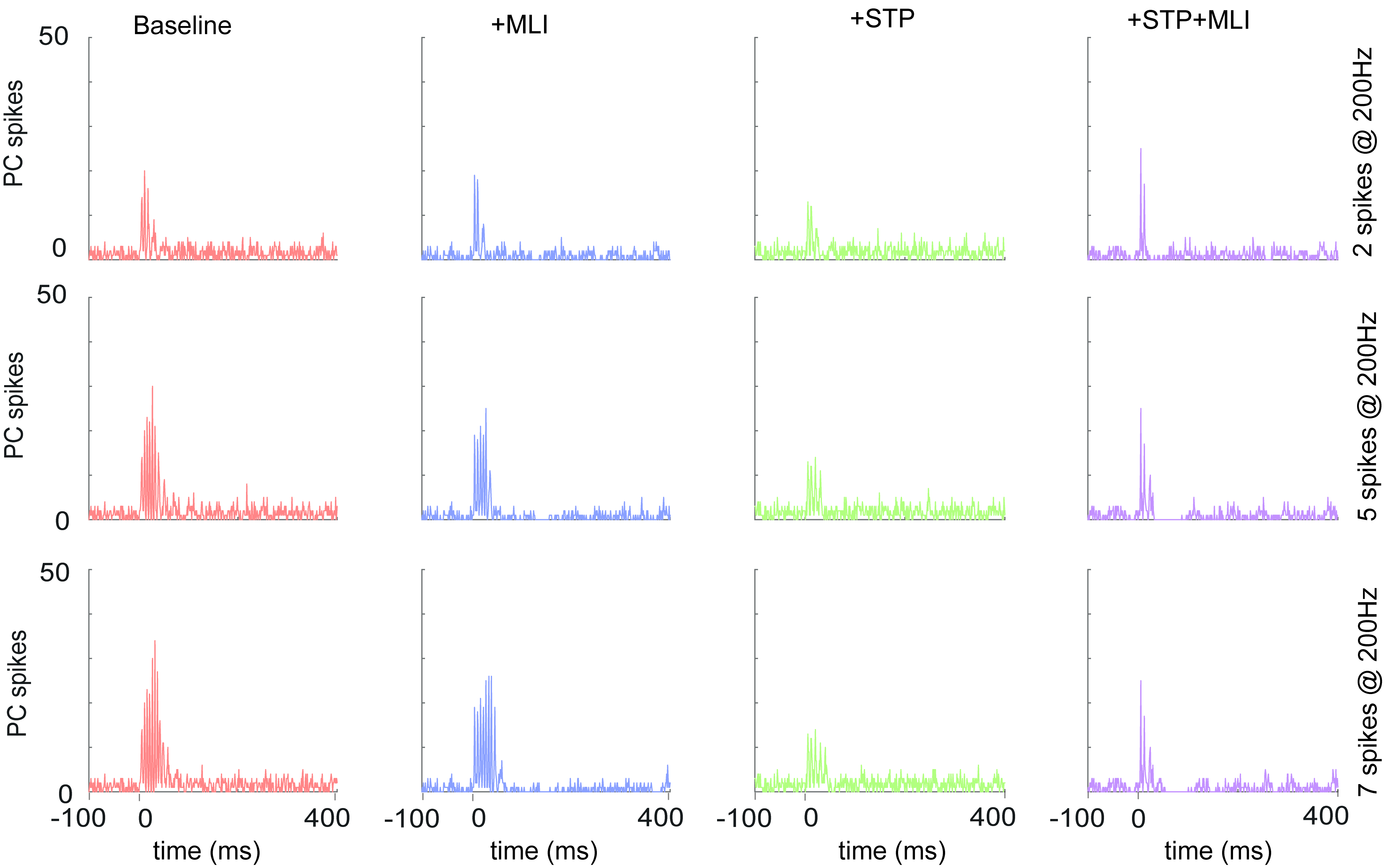

Supplement: S9 Fig — PC population firing rate under bursts of 200 Hz with 2, 5, and 7 spikes in four conditions. The background Poisson stimulation is 20 Hz. (TIF) [file pcbi.1008670.s009.tif]
