# Supplementary material for: Modulation of the dynamics of cerebellar Purkinje cells through the interaction of excitatory and inhibitory feedforward pathways
Source: PLoS Comput Biol. 2021 Feb 10;17(2):e1008670. doi: 10.1371/journal.pcbi.1008670 (PMC7909957; doi:10.1371/journal.pcbi.1008670)
Supplement: Attachment — Submitted filename: PCB_response.pdf [file pcbi.1008670.s011.pdf]

PLOS Computational Biology (PCOMPBIOL-D-20-00987)

Dear Editor and Reviewers:

First of all, we would like to express our gratitude to you for carefully reading our manuscript and giving us constructive suggestions. We have carefully considered your comments, and revised our manuscript in light of your remarks and suggestions. You are generous sharing both your time for carefully reading our manuscript and expertise facilitating our work. Thank you all for your comments during the revision of our manuscript.

The following are point-to-point responses to the reviewers' comments. The reply is arranged in Q's and A's. We first quote your comments, and then give our answers after each of your questions.

Replies to the Comments from Editors:

From reading the reports, all the points raised seem reasonable and should be addressed in a reworked manuscript. In particular, the reviewers were concerned about clarity of the main message, as well as model choices. A revised manuscript should have substantial effort devoted to robustness tests and/or motivation of the parameters.

We have carefully revised our manuscript in light of the comments provided by the reviewers. We revised our writing significantly to improve the clarity of the main message and model choices, added more analysis with additional parameter settings for robustness tests (Fig.S3, Fig.S6), replotted Fig.1 (with Fig.S2) for motivation of the parameters, revised Fig.6 for better explanations, and extended our Discussion Sec.

We provided a version of the revised manuscript with highlights denoting where the text has been revised (in red) for easy reviewing. Please find the marked PDF file uploaded as Related Manuscript '**PC_network_marked-up.pdf**'. All the responses below indicated by line numbers refer to the marked PDF file rather than the clean copy PDF file.

Thank you very much.

Replies to the Comments from Referee #1:

There has been increased interest in understanding the role of synaptic dynamics such as short-term plasticity (STP) in shaping network activity in the cerebellar cortex. The authors here have investigated the interaction of such STP at a particular synapse (parallel fibre-purkinje cell) along with feedforward inhibition, in shaping both the response of individual Purkinje cells as well as their network response. While the authors have systematically explored various downstream effects for different parameter regimes, the reviewer has some concerns regarding the specific protocols used, as well as the default model regime used.

Major Points:

Q-I-1: The authors use U, a parameter that describes synaptic efficacy/probability of release, and along with timescales of recovery and facilitation, determines whether the synapse is facilitating or depressing. Given that this parameter is changed for most effects explored, knowing how this parameter changes the shape of STP is critical to the reader for understanding/appreciating the results. In light of this, it would be important to help the reader know what ranges of U correspond to facilitation versus depression.

- *For example, the changes in unitary EPSP and train of EPSPs for different U, as shown in Fig 9, could be included in Fig 1, to motivate the reader.*
- *Additionally, a similar analysis should be shown for the MLI-PC synapses which are modelled to have STP (but never discussed), but may have different profiles as the net facilitation/depression depends on the timescales (t-fac and t-rec)*

A-I-1: Thanks for the suggestions. We have updated Fig.1, together with Supplemental Fig. S2, as suggested by the reviewer, with the profiles of unitary and EPSP/IPSP and a train of EPSPs/IPSPs to better motivate the reader.

A similar analysis for the MLI-PC synapses was also added. Indeed, we included the STP for MLI-PC synapses in our model. We now add additional analysis with blocking the STP of MLI-PC synapse (see Supplemental Fig. S3 and Supplemental Fig. S6), i.e., there are no short-term dynamic updates when STP is muted. Our results are robust to the parameters of the MLI-PC STP (see the A-I-2 response below for more discussion). We added these new analyses and revised the text (see line 126-146 colored in red in the marked PDF file).

Q-I-2: The authors have cited several papers used to constrain the model synaptic parameters, however some of the model choices are not explained, especially where they are at odds from previous publications.

- *Specifically, a similar study published in 2019 [Grangeray-Vilmint et al, **ref 22**] that performed slice recordings and modelling to estimate STP at both GC-PC and MLI-PC synapses had estimated different values of **U** from their experimental data. They estimated GC-PF to be mostly facilitating, whereas GC-MLI and MLI-PC synapses to be mainly depressing (also see: ref [23], and Bao et al, 2010: Target-Dependent Feedforward Inhibition Mediated by Short-Term Synaptic Plasticity in the Cerebellum). In contrast, this study uses a different range of U (higher U for GC-PC making them more depressing, lower U for MLI-PC making them less depressing). It would be helpful if the authors discuss these differences, or show that the behaviours are still consistent (for example, compensated by difference in timescales?)*
- *Importantly, this difference in STP profile also leads to different downstream consequences. In the current study, over longer input trains/GC bursts, inhibition would start to dominate because of stronger depression of the excitatory GC-PC synapses. This may contradict the experimental data presented in ref 22, which had increased response for longer GC bursts, which was explained by strong depression of MLI-PC synapses. Given that the default regime adopted in the current study (U-exc = 0.4 and U-inh = 0.1) likely have the opposite profile to previously published data/models, further justification of these choices may be required.*

A-I-2: Thanks for pointing this out. Indeed, U is an important parameter for STP. We have shown the effect of U on the results as in Fig. 9-10 with a wide range of values besides our default values (U-exc=0.4 for depression and U-inh=0.1 for facilitation), and we found that our

results are consistent across different settings of the parameter U. To make this point more clearly, we added additional analysis according to the suggestions of the reviewer as below.

As noted by the reviewer, some experimental studies have suggested the possible values of the parameter U, which are different from our default values. To explain that our results are robust to the parameter setting, we added some analysis with an opposite set of values (U-exc = 0.08 and U-inh = 0.2). Similar to Ref. 22 and what the reviewer suggested, U-exc = 0.08 is for facilitating GC-PC synapses and U-inh = 0.2 is for depressing MLI-PC synapses. We used these opposite values to show two key observations of our study. 1.) Related to Fig. 2 and Supplemental Fig. S3, the profiles of gain change of the PC firing are similar. 2.) Related to Fig. 7 and Supplemental Fig. S6, the network synchronization and pause responses are also similar. These examples, together with Fig. 9-10, confirm that our results are not depending on the exact setting of the STP on GC-PC and MLI-PC synapses, whether facilitation or depression. We have included these results in our revised manuscript (see line 168 and line 276).

Q-I-3: Burst protocol used in Figure 7-10 consists of adding burst of spikes to the entire GC population. Could the authors discuss the feasibility of such a protocol? In contrast, the protocols used previously (eg ref 22) consist of bursts in single GCs, and measure the efficacy of transmission of these inputs. Furthermore, the synchrony observed in PC population (Fig 9, 11) may thus be a direct result of this massively coincident GC inputs. Additionally ref 45, cited on line 284, does not suggest such coincident GC input as a source of synchronization, but ephaptic coupling via extracellular currents. Is there an alternative reference for this synchronized GC activation?

A-I-3: Thanks for pointing this put. The burst protocol used in our study is to characterize the synchronization of PCs at the network level. As showed in Ref. 22 and mentioned by the reviewer, bursts in single GCs are a useful experimental protocol for studying transmission of these inputs to downstream PCs. Physiologically, it is not possible to have bursts in the entire GC population. However, it is possible to have massively coincident GC inputs, given the GC population is large and dense in the granular layer. With current experimental techniques, delivering such a massive protocol could only be possible for a few tens neurons, for example, optical stimulation. The reason we use this protocol is to simulate the effect of a large number of coincident inputs, which is a typical protocol for modeling studies [1], and see how they are propagated to PCs. We revised our text to make it clear (see also line 415).

[1] Stimulus-dependent state transition between synchronized oscillation and randomly repetitive burst in a model cerebellar granular layer. Honda, T.; Yamazaki, T.; Tanaka, S.; Nagao, S. & Nishino, T. PLoS computational biology, 7, e1002087 (2011)

Indeed, as mentioned by the reviewer, Ref. 45 shows that ephaptic coupling can contribute to the prevalent synchronization of nearby PCs, so we revised the text regarding this citation. Ref. 45 also includes the discussion about the possible source of synchronization from GCs (see references within Ref.45, for example, [2]). As discussed in [2], GC synchronization is a general phenomenon that could be due to the Golgi cells [3-4]. However, the mechanisms of PC synchronization are still under debate and could be due to a combination of multiple factors [5]. We added these discussions in the Discussion Sec. (see line 415).

[2] Temporal Organization of Activity in the Cerebellar Cortex: A Manifesto for Synchrony. Isope, P.; Dieudonne, S. & Barbour, B. Annals of the New York Academy of Sciences, 978, 164-174 (2002)

[3] Synchronization of golgi and granule cell firing in a detailed network model of the cerebellar granule cell layer. R Maex & E De Schutter. J Neurophysiol 80(5):2521-37 (1998)

[4] Parallel Fibers Synchronize Spontaneous Activity in Cerebellar Golgi Cells. Bart P. Vos, Reinoud Maex, Antonia Volny-Luraghi and Erik De Schutter. Journal of Neuroscience. 19(11) (1999)

[5] Spatiotemporal firing patterns in the cerebellum. Chris I. De Zeeuw, Freek E. Hoebeek, Laurens W. J. Bosman, Martijn Schonewille, Laurens Witter & Sebastiaan K. Koekkoek. Nature Reviews Neuroscience 12, 327–344 (2011)

Q-I-4: In continuation with the previous point, the burst protocol used here leads to depressed PC responses for longer trains, due to the cooperative facilitation of MLI-mediated inhibition and depression of GC-PC excitation. This is in contrast to experimental data in ref 22. Could the authors fully explain this discrepancy (in light of the additional difference in protocols and synaptic STP profiles)?

A-I-4: Thanks for the suggestion. We have included additional analysis using the parameters similar to Ref. 22 (see Supplemental Fig. S6 and our response A-I-2 above). Our results are consistent within a wide range of parameter settings, and not depending on the specific profiles, facilitation or depression, of STP.

Q-I-5: In Fig 6, temporal profile of PC response is evaluated with 10 Hz stimulations that are lower than most ranges used elsewhere in the paper. Could the authors discuss this choice, especially given that the synaptic dynamics can switch from facilitating to depressing at low to high frequencies? Further, if the aim is to explore reliability of PC spike timings, some of the more direct analyses in Fig S4 may be more relevant than mean ISIs currently included in Fig 6, which only convey the firing rate rather than temporal aspects?

A-I-5: Thanks for the suggestion. Here we used 10 Hz Poisson stimulations in Fig. 6 to visualize the individual temporal profiles of PC response under different settings (see also in A-I-2 with different profiles of STP). It is true that more direct analyses are more suitable for better explanations in Fig.6, so we have updated Fig. 6 as suggested by the reviewer to include direct analysis.

Q-I-6: The authors suggest that STP enhances reliability of PC spike timings (in Fig 6, S3-4) which may seem intuitively at odds with decreased PC synchronization observed with STP (fig 10). Could the authors discuss the difference in protocols, eg input frequency (10 Hz vs 50-300Hz) used to quantify these response features?

A-I-6: Sorry for the confusion. We have updated the text and Fig.6 to make them clear. Fig. 6 is more focusing on explaining the role of combined STP+MLI for PC spike timings on the single cell level, which then was followed by Fig.7 and other results on the network level. We

only used 10 Hz of Poisson stimulation for illustration as explained in A-I-5. The burst stimulation is delivered on top of background Poisson stimulation (noise). We manipulated the parameters of the burst protocol, e.g., 10 spikes firing at different frequencies and the same frequency with different spikes (Fig.8-11) to see the effect on the network.

Minor points:

Q-I-7: Could the authors clarify (perhaps in Methods) what turning off STP means, especially as they change U to different levels in Fig 3, in the absence of STP. Specifically, does it mean that the synaptic resource (R) and release (u) are held fixed (at 1 and U respectively), rather than following the dynamics specified? Then changing U would effectively scale the excitatory conductance. Furthermore, could the authors clarify whether STP at other synapses was also removed, or only at GC-PC synapses?

A-I-7: Yes, the reviewer is right here. Turning off STP means that the variables R and u are held fixed (R=1, u=U) without dynamic updates in response to spikes. We added this description in Methods (see line 511) as well as in the beginning (see line 150). Through our manuscript, we only manipulate the STP at GC-PC synapses when turning on and off STP. All other synapses are dynamic as described by the rule of STP. In the new analysis (Fig. S3 and Fig. S6), we also muted the STP (R=1, u=U) of MLI-PC synapses.

Q-I-8: It would be helpful if justification for the number of neurons of each cell class, and the number of activated cells could be discussed in methods, given that the anatomical expansion ratios are different?

A-I-8: Granule cells are known as the most numerous neurons, and other cerebellar cells are relatively fewer. However, there is no detailed confirmation about the number of neurons of each cell class as well as the number of synaptic connections between cells (perhaps the clear example is that each GC receives about 4 mossy fibers). Recent studies using 2-photo imaging on a population of GCs suggest that GCs are encoding more types of information than motor information, the number of activated GCs is considerably more significant, and the spatial organization of granule cell activity can be examined (see [1], for example). In this study, we only use a relatively small network to study a variety of firing modes. But the reviewer is right, future modeling work has to take into account the newest experimental observation, and we hope to address these questions in our future work.

[1] Cerebellar granule cells: dense, rich and evolving representations. A Badura, CI De Zeeuw. Current Biology, 27(11) R415-R418 (2017)

Q-I-9: Line 168-170, and Discussion line 411-413: mentions multiplicative scaling of I-O curves with MLI-dependent inhibition but it actually looks like offset (additive change) in fig 2c. This is also quantified as no gain change in –STP +/- MLI in Fig 2D.

A-I-9: Thanks for pointing this out. We have corrected this point.

Q-I-10: Line 148: Introduces concept of high dimensional representations at GCs. However these are mostly postulated, and not experimentally verified/established. It would be helpful to cite the main theoretical papers that formulated this hypothesis. Eg: Marr 1969, Albus 1971, Cayco-Gajic and Silver 2019.

A-I-10: Indeed, these references are helpful, and we added them to our revised text.

Q-I-11: Fig 4&5: Phase shifts – It is unclear how to interpret the relatively modest changes in phase shifts with input frequency, with different synaptic parameters. Example, Could variability (across simulations) or effect size comparsion be added?

A-I-11: The relatively modest changes in phase shifts examined here are represented by a population of PCs. There is a variation of parameter setting in neurons and synapses in the network model, and we also used a Poisson process to sample input spikes modulated by the sinusoidal firing rate over multiple cycles of frequency, so these are equivalent to showing the variability across simulations. We make this clear in our revised text.

Q-I-12: Line 64: Possibly wrong reference inserted, should be ref 9 instead of ref 10?

A-I-12: Thanks for pointing this out. We have corrected the citation and carefully revised all the citations.

Q-I-13: Figure 1D: Notes 3 example PC traces as well as FR curves. However, it is unclear there are 3 highly overlapping curves. If all cells have nearly identical curves in the absence of inhibition/STP, can a single example/average curve be plotted instead? Or the legend could clarify there are 3 overlapping curves?

A-I-13: Thanks for the suggestion. Here we want to show the slight difference in PC responses induced by randomization of parameters in the model. We now moved this plot panel to Supplemental Fig. 1.

Q-I-14: Fig 3B: Legend says PC firing but y-axis label seems to indicate it is net excitatory conductance?

A-I-14: We have corrected it.

Q-I-15: Figure 5c: For each small panel, indicate what is the frequency range (0 to 30 from top to down, or 30 to 0?) Is it the same as Fig 4?

A-I-15: Thanks for pointing this out. Yes, they are 30-0 Hz from top to down. We have updated Fig. 4 and Fig. 5 with this label.

Replies to the Comments from Referee #2:

Reviewer #2: In the cerebellum, Purkinje cells (PCs) receive feedforward inputs mainly from two neural pathways: the first one is the feedforward excitatory pathway of parallel fibers from granule cells (GCs) to PCs; the second one is the feedforward inhibition pathway from GCs,

via molecular layer interneurons (MLIs), to PCs. This manscuript [PCOMPBIOL-D-20-00987] have systematically invesigatted how the dynamics of Purkinje cells (PCs) are jointly modulated by these two feedforward pathways. To this end, the authors established a simplified PC network with short-term plasticity, in which the firing dynamics of GCs, PCs and MLIs are simulated with the integrate-and-fire neurons, and different stimulation protocols of mossy fiber inputs are used to drive the network activity. Briefly, the authors showed that the interaction of feedforward pathways of excitation and inhibition, together with synaptic short-term dynamics, can dramatically affect the PC responses that consequently change the network dynamics of the PC circuit. The topic of this manuscript is quite interesting, and the manuscript is well organized and written. I only have a few comments and suggestions that the authors may consider during revision.

Comments and suggestions:

Q-II-1: Although the topic of this study is interesting, the title of this manuscript is inappropriate. Indeed, it does not contain any word related to the cerebellum, making the title seems to be too general. Please revised to clearly explain the research.

A-II-1: Thanks for the suggestion. We have changed the title as: Modulation of the dynamics of cerebellar Purkinje cells through the interaction of excitatory and inhibitory feedforward pathways.

Q-II-2: I strongly suggest the authors focus on the PC network in this study, and several general sentences should be removed from the manuscript, in particular in the abstract and introduction.

A-II-2: Thanks for the suggestion. We have revised the related text by removing the non-very-relevant general sentences, and putting the relevant ones into the concrete context of the cerebellum and PCs (see the revised abstract and introduction).

Q-II-3: In Fig 3, the authors showed that GC-PC synaptic STP with the aid of MLI inhibition can significantly enhance gain modulation of PC firing dynamics. This is one key finding of this manuscript because gain control of cortical neurons are believed to be highly associated with their neuronal computations. I suggest the authors to compare their modeling findings with experimental results on PCs, or at the very least I would expect you to further discuss it.

A-II-3: Thanks for the suggestion. Indeed, this is an important point. We revised our discussion section and added more discussion about this point and the relevant experimental results, see the text (line 390-399) in the Discussion Sec.

Q-II-4: In this study, both the conduction delay on fibers and the synaptic transmission delay are not considered in the PC network. In fact, transmission delay might play important roles in controlling the dynamics of the PC networks. In particular, it might significantly impact the PC firing phase and contribute to the phase shift in neural dynamics in response to time-varying inputs.

A-II-4: Thanks for pointing this out. We do have synaptic delays included in our model. Now we have added this information in the Methods part (see line 512). For all synapses, the delay is set as 1 ms. For MLI-PC inhibition synapses, delays are more heterogeneous with a Gaussian distribution (mean as 1ms and SD 0.2 ms). Together with other randomizations of the model parameters, our model presents robust results whilst including random effects. Our model with the code will be released to the public as part of open science.

Q-II-5: In the cerebellar granular layer, there are several Golgi cells. The GoCs inhibit the numerous GCs through sagittal branching of their axons and, in addition, they are also connected together by gap junctions. Recent modeling studies have shown that GoCs may play roles in the emergence of granular oscillations. Although the authors did not include the Golgi cells in their PC network model, I still suggest them to further discuss the possible regulations of GoCs in the dynamics of PCs co-modulated by feedforward GC-PC and MLI-PC pathways.

A-II-5: Indeed, GoCs have been suggested to play an important role in the dynamics of the granular layer, particularly, contributing to the network dynamics of GCs as synchronization and oscillation. Thus, GoCs could contribute to the modulation of PC firing dynamics. We added more discussion about this point (see line 472-478, see also the response A-I-3) with a new subsection “Limitations” in the Discussion Sec. As there is no direct experimental evidence, as far as we know, but some potential hints [1-3] on how GoCs regulate PC dynamics, we agree with the reviewers that it is an important topic for modeling studies. Thanks for the suggestion, we are planning to conduct this part in detail and would like to report our results with a further in-depth investigation.

[1] The cerebellar Golgi cell and spatiotemporal organization of granular layer activity. D'Angelo, E.; Solinas, S.; Mapelli, J.; Gandolfi, D.; Mapelli, L. & Prestori, F. Frontiers in Neural Circuits. 7:93 (2013)

[2] Different responses of rat cerebellar Purkinje cells and Golgi cells evoked by widespread convergent sensory inputs. Holtzman, T., Rajapaksa, T., Mostofi, A. & Edgley, S. A. The Journal of Physiology, 574, 491-507 (2006)

[3] Ablation of cerebellar Golgi cells disrupts synaptic integration involving GABA inhibition and NMDA receptor activation in motor coordination. Watanabe, D.; Inokawa, H.; Hashimoto, K.; Suzuki, N.; Kano, M.; Shigemoto, R.; Hirano, T.; Toyama, K.; Kaneko, S.; Yokoi, M.; Moriyoshi, K.; Suzuki, M.; Kobayashi, K.; Nagatsu, T.; Kreitman, R. J.; Pastan, I. & Nakanishi, S. Cell. 95, 17-27 1998

Q-II-6: There are too many references in the manuscript but several important references are missing. The authors may be interested in the following recent published papers: SK Sudhakar et al., PLoS Comput. Biol., 2014 & 2015; Y Zang & E De Schutter, Front. Syst. Neurosci., 2019.

A-II-6: Thanks for the suggestion. These relevant references have been properly cited. We also carefully revised our reference list to make it concise.

Replies to the Comments from Referee #3:

Reviewer #3: This paper employs a basic model of a cerebellar circuit to address how the interaction of the feed-forward inhibitory pathway and the short term plasticity of the feed-forward excitatory pathway from granule cells control Purkinje cell output and associated circuit activity.

While I consider that this is an important subject in the context of cerebellar circuit dynamics, the paper fails to deliver a clear message and convincing results regarding the generality of the model beyond the specific choice of description level and parameters.

Here are a few suggestions to improve the paper:

Q-III-1: Authors could emphasize from the beginning the model description level: point neurons implemented with an integrate and fire model. Nothing wrong about this choice, but of course limitations arise from how the intrinsic neuron dynamics integrates the combined effect of the two different pathways. Authors could further discuss how a morphological distribution of the inputs and more complex active channel description in PC cells could build up in the mechanisms described. Limitations and generality of the results with respect to the chosen approach should be discussed in further detail.

A-III-1: Thanks for the suggestions. We have revised the text to make it clear that neurons are modeled as point neurons. In addition, now we add a new paragraph in Discussion Sec "Limitations" for discussion of morphological effects and active channels as intrinsic mechanisms (line 457-471).

Q-III-2: Beyond noise, authors do not provide a validation of the robustness of the described phenomena as a function of the parameters of the neuron model, just the dependence of the dynamic synapse parameters is assessed. This can be easily addressed in more simplified models as the one used in this study. Furthermore, no justification of the parameters chosen is provided, in particular time constants.

A-III-2: Thanks for the suggestion. We have added more description in Methods about the justification of the parameters for neurons. As the main focus of this work is about neural pathways of synapses, we only considered the synaptic dynamics and their parameters. The neuronal parameters, as part of intrinsic properties (as mentioned in A-III-1), are not investigated. But we agree that they are important. We have included a new Discussion Sec. "Limitations" for discussion of the issues unaddressed in this work.

Q-III-3: The role of climbing fibers in only hinted in the discussion. it should at least be introduced at the beginning of the manuscript and a justification of why they are left out could be provided.

A-III-3: Thanks for the suggestion. Here we only focus on the direct neural pathways from GCs, via MLIs, to PCs, thus we did not consider the effect of climbing fibers. As mentioned in A-II-1, the role of climbing fibers could be addressed when the PC morphology is taken into account, since it is generally believed that climber firers target different parts of PC dendrites than parallel filers from GCs. Thus, it could be the 3rd factor to modulate PCs, besides two neural pathways addressed here. Now we added more discussion in Discussion sec. “Limitations” to address the role of climbing fibers (line 460 about morphology and 479 about the role of climbing fibers).

Minor issues:

Q-III-4 Please clarify what stimulation at -60, 70, 0 mV is in Fig. 1B

A-III-4: The current clamp is used in experiments so that current is injected to maintain the membrane potential at different values, such as clamped at -60, -70, 0 mV. For clarity, we moved the description to the Methods part and add more explanation there.

Q-III-5 Line 128, please justify the choice of the model from Ref. 20.

A-III-5: The models of GCs and their synapses with short-term plasticity are based on Ref. 20, where experimental data were used to constrain the model. We revised the text to make it clearer in Methods.

Q-III-6 Please justify the choice of all parameters in section IV and table I.

A-III-6: We have revised the text to make them clearer in Methods. The neuronal parameters in Table I were based on several previous studies about GCs, MLIs and PCs (line 501). Synaptic parameters were also based on previous studies to get default values (line 515). We symmetrically varied the synaptic parameter values as part of neural pathways.

Q-III-7 Please fix discrepancies between labels and figure caption in Fig. S7

A-III-7: Thanks, we have corrected it.
