# Supplementary material for: Modulation of the dynamics of cerebellar Purkinje cells through the interaction of excitatory and inhibitory feedforward pathways
Source: PLoS Comput Biol. 2021 Feb 10;17(2):e1008670. doi: 10.1371/journal.pcbi.1008670 (PMC7909957; doi:10.1371/journal.pcbi.1008670)
Supplement: Attachment — Submitted filename: PCB_response.pdf [file pcbi.1008670.s012.pdf]

PLOS Computational Biology (PCOMPBIOL-D-20-00987R1)

Dear Editor and Reviewers:

First of all, we would like to express our gratitude to you for carefully reading our manuscript and giving us constructive suggestions. We have carefully considered your comments, and revised our manuscript in light of your remarks and suggestions. You are generous sharing both your time for carefully reading our manuscript and expertise facilitating our work. Thank you all for your comments during the revision of our manuscript.

The following are point-to-point responses to the reviewers' comments. The reply is arranged in Q's and A's. We first quote your comments, and then give our answers after each of your questions.

Replies to the Comments from Editors:

The reviewers found the manuscript to have improved, however there still remain some concerned outlined by Reviewer 1. These points seem reasonable and should be addressed in your next submission.

We have carefully revised our manuscript in light of the comments provided by Reviewer 1. We revised our writing to improve the clarity of the text, added more analysis using Hill functions to fit data and new plots with lower values of W_MLI-PC (Fig.2 and Fig.S3), and add new plots of Gexc and R (Fig. S7), and revised our Discussion Sec.

We provided a version of the revised manuscript with highlights denoting where the text has been revised (in red) for easy reviewing. Please find the marked PDF file uploaded as Related Manuscript '**PC_network_marked-up.pdf**'. All the responses below indicated by line numbers refer to the marked PDF file rather than the clean copy PDF file.

Thank you very much.

Replies to the Comments from Referee #1:

The manuscript "Modulation of the dynamics of cerebellar Purkinje cells through the interaction of excitatory and inhibitory feedforward pathways" uses reduced network modelling of the cerebellar cortex to study how the interaction of excitatory and inhibitory feedforward pathways affect response of cerebellar Purkinje cells. The reviewer acknowledges the substantial efforts of the authors to include further simulations, analyses and parameter exploration, and the consistency of these results with previous findings strengthen the paper. However, there are some questions raised by the apparent robustness of the observations to the precise form of STP, that would need to be discussed, as well as some suggestions on improving the clarity of the text and methods.

Major Points:

Q-I-1: Major comments:

1) Figure 2 characterizes the change in gain of PC Input-Output function, as the change in average slope. However, the I-O functions are highly non-linear (with a saturating non-linearity). The gain calculation in reference [28] fit a convolution of Hill function and exponential to the measured I-O. Can the authors please clarify (in methods and main text) if they also fit a similar non-linear function, and refer to the slope parameter in that model, and not to a linear fit to the simulation data?

Further, in Figure 2B and 2C, can the legend clarify what the lines and scatter are (fits versus simulation?) Also, to support the statement that "MLI-mediated inhibition introduces an additive shift" on line 166-167, it seems necessary to additionally show changes in offset, along with gain in Figure 2D?

A-I-1: Sorry for the confusion, and thanks for the suggestion. We used a double exponential function, with rising and decay terms, to fit simulation data. However, as the reviewed suggested, now we replaced it with a Hill function as in Ref. [28], and replotted Fig. 2 and Fig.S3, so that the results can be fitted well with Hill functions. We also computed both gain and offset as in Ref. [28] and added the results into the figures. We updated figure legends to make them clear for fits (lines) and simulation data (dots).

We updated our Methods section and revised the text about this part (red text in the marked PDF file).

Q-I-2: For Supplementary Figure 3A, the black and cyan lines (without STP at MLI-PC synapses) seem to have no PC output – is that because of inappropriate scaling of inhibitory conductances? Can that be recovered by reducing the weight of MLI-PC synapses?

It is also difficult to compare the results in S3A and S3B directly because fixing U (in no STP cases) at different values also changes scaling of synaptic currents? If the claim is that the results are robust to those parameters, perhaps the quantification of gain/offset change can be added to the figure, or highlighted as the corresponding column in Figure S4B? Further, can the authors discuss (in addition to line 174-176) why the two curves end up looking similar in the two scenarios – is it mainly that for all values of U, synapses become depressing at high enough stimulation frequency (resource variable R goes near 0)? This suggests that there is still a difference at lower frequencies – of facilitation versus depression – which can be seen in Figure 3B?

A-I-2: Yes, in Fig.3A, we used a different pair of STP parameters, compared to Fig.3B, without changing the weight of MLI-PC synapses, which made PCs no output. Now, we added a few more plots (Fig.S3B) with reduced weights of MLI-PC synapses, and there are firing outputs as a consequence of weaker inhibition. We also added the quantification of gain/offset changes to Fig.S3.

Yes, indeed, Fig. S3 is to show whether detailed profiles of facilitation or depression in STP could have an effect on gain control, since some experimental results, as discussed in the last response, reported different profiles of STP facilitation or depression. As facilitation needs lower values of U, whereas depression for higher values of U, fixed U values changes scaling of synaptic currents, as well as profiles of STP. However, STP also depends on input frequency, so that lower frequencies show different behaviour than higher frequencies, which has been further studied in Fig.3. We now added more discussion about this point, in line with what reviewer discussed (line 182).

Q-I-3: Line 390-399: Cited reference [51] is mentioned as showing linear IO response of PCs without MLI-mediated inhibition (which is not entirely consistent with the results perhaps, Fig3A for example). Further, it is unclear how this links to the effect of turning off STP at GC-PC synapses (line 391-392). Is it mentioned as an alternative mechanism for generating nonlinear IO responses? The experimental recordings did not eliminate STP and still observed linear response curves, which is again not entirely consistent with these modelling results. Although the photolysis of glutamate experiment do bypass synaptic dynamics, the electrical stimulation of parallel fibres also produced linear PC synapses. As these stimulations are not necessarily linearly related to input frequency/strength, it may be important to mention this caveat to explain the discrepancy between the data and model, or to exclude this reference/discussion altogether.

A-I-3: Thanks for the note. Indeed, our modelling results are not entirely consistent with experimental results of Ref. [51]. As the reviewer noted here, experimental conditions and protocols used in Ref. [51] might be true in some circumstances. However, it is more general demonstrated in the neurons of Cerebellum and other brain areas, nonlinear IO responses are more visible. Thus, we revised our discussions exclude this part of discussion (line 395)

Minor points:

Q-I-7: Minor comments:

1) As was mentioned in the previous round, the default parameters used in this study for Uexc at GC-PC and Uinh for MLI-PC differ from published experimental results and previous modelling (see refs: 18, 19, 27, 36). In particular, in figure 1B, although kinetics of single EPSP are fitted, as well as STP for GC-MLI synapses, the STP dynamics for GC-PC synapses, (and MLI-PC), which are the main objects of study here, are specifically not fitted. Although some results are robust to the parameter choice (partly because all synapses become depressing at high frequency stimulation), it may be better to explicitly mention that other aspects of PC response may still vary for different STP dynamics.

A: Thanks for the note. We have revised the text to explicitly mention that PC responses vary for different STP dynamics, as part of the change of STP parameters.

2) For Figure S4B, can the legend clarify whether the change in gain was compared to a single fixed IO curve, or to the corresponding baseline for each case (for example for each Uexc, it is compare to an IO with the same U with and without its dynamics?)

A: Sorry for the confusion. We have revised the figure legend. The change in gain was compared to the baseline for each case.

3) Line 129: " a single MLI of inhibition" -> "inhibition from a single MLI"

A: Thanks, we changed it.

4) Line 193-194 about gain control being maintained is unclear. Do the authors mean the change in excitatory conductance with stimulation frequency is similar across different values of Uexc (however there is a large difference between them for 10-50Hz inputs, and reduced difference for 60-140Hz inputs).

A: Sorry for the confusion. We now revised the text in line the reviewer's suggestion: "….The gain change is well displayed, in particular with larger difference for 10-50 Hz GC inputs, whereas less difference at higher frequencies due to saturation of short-term dynamics (Fig3B, bottom)…"

5) Line 252-255: The cited reference [35] shows that regularity of spiking is not an important feature for the fast "rate" coding by Purkinje cells. Thus it may be inappropriate to cite that to suggest that differences in ISI and CV (dependent on STP and inhibition) change information transmission by PCs, as the claim in the reference is opposite.

A: Sorry for the confusion. We are not citing the conclusions of Ref. [35]. We mean that we used these similar measures to quantify spike patterns, which were also used and defined as in Ref. [35]. To avoid the confusion, we revised the text by removing the sentence and moved this citation to the Methods part: spike train analysis.

6) Figure 7 and S6: Although the pause response doesn't depend on precise U, is it due to the specific stimulation frequency (i.e. at 100Hz – 300Hz), all GC-PC synapses become effectively depressed? Plotting the evolution of Gexc, R variables may be insightful.

A: Thanks for the insightful suggestion. We added a new figure Fig.S7, where the evolution of Gexc and R is shown.

7) Line 285: Consisting -> Consistent

A: Thanks, we have corrected it.

8) Line 287-288: It is unclear what the main conclusion of Fig 8A and FigS7 is. Can the authors help the reader focus on what is meant by "different PC dynamics"?

A: We now added more text on this part (line 289).

9) Line 409: depression -> depressing;

A: Thanks, we have corrected it.

Reviewer #3: ***No:*** *The authors mention in the response to the reviews that "Our model with the code will be released to the public as part of open science." but no link is available in the manuscript.*

A: We will add the link at the dedicated location of Plos Computational Biology:

Data Availability: All code used to generate the results in this paper will be available on the GitHub (https://github.com/jiankliu).
