# Supplementary material for: Modulation of the dynamics of cerebellar Purkinje cells through the interaction of excitatory and inhibitory feedforward pathways
Source: PLoS Comput Biol. 2021 Feb 10;17(2):e1008670. doi: 10.1371/journal.pcbi.1008670 (PMC7909957; doi:10.1371/journal.pcbi.1008670)
Supplement: Attachment — Submitted filename: Tang_etal_July2020.docx [file pcbi.1008670.s010.docx]

Review for **PCOMPBIOL-D-20-00987**

**Title:** 'Modulation of neural dynamics through the interaction of excitatory and inhibitory feedforward pathways'

There has been increased interest in understanding the role of synaptic dynamics such as short-term plasticity (STP) in shaping network activity in the cerebellar cortex. The authors here have investigated the interaction of such STP at a particular synapse (parallel fibre-purkinje cell) along with feedforward inhibition, in shaping both the response of individual Purkinje cells as well as their network response. While the authors have systematically explored various downstream effects for different parameter regimes, the reviewer has some concerns regarding the specific protocols used, as well as the default model regime used.

Major Points:

1. The authors use ***U***, a parameter that describes synaptic efficacy/probability of release, and along with timescales of recovery and facilitation, determines whether the synapse is facilitating or depressing. Given that this parameter is changed for most effects explored, knowing how this parameter changes the shape of STP is critical to the reader for understanding/appreciating the results. In light of this, it would be important to help the reader know what ranges of U correspond to facilitation versus depression.
   - For example, the changes in unitary EPSP and train of EPSPs for different U, as shown in Fig 9, could be included in Fig 1, to motivate the reader.
   - Additionally, a similar analysis should be shown for the MLI-PC synapses which are modelled to have STP (but never discussed), but may have different profiles as the net facilitation/depression depends on the timescales (t-fac and t-rec)
2. The authors have cited several papers used to constrain the model synaptic parameters, however some of the model choices are not explained, especially where they are at odds from previous publications.
   - Specifically, a similar study published in 2019 [Grangeray-Vilmint et al, **ref 22**] that performed slice recordings and modelling to estimate STP at both GC-PC and MLI-PC synapses had estimated different values of ***U*** from their experimental data. They estimated GC-PF to be mostly facilitating, whereas GC-MLI and MLI-PC synapses to be mainly depressing (also see: ref [23], and Bao et al, 2010: Target-Dependent Feedforward Inhibition Mediated by Short-Term Synaptic Plasticity in the Cerebellum). In contrast, this study uses a different range of U (higher U for GC-PC making them more depressing, lower U for MLI-PC making them less depressing). It would be helpful if the authors discuss these differences, or show that the behaviours are still consistent (for example, compensated by difference in timescales?)
   - Importantly, this difference in STP profile also leads to different downstream consequences. In the current study, over longer input trains/GC bursts, inhibition would start to dominate because of stronger depression of the excitatory GC-PC synapses. This may contradict the experimental data presented in ref 22, which had increased response for longer GC bursts, which was explained by strong depression of MLI-PC synapses. Given that the default regime adopted in the current study (U-exc = 0.4 and U-inh = 0.1) likely have the opposite profile to previously published data/models, further justification of these choices may be required.
3. Burst protocol used in Figure 7-10 consists of adding burst of spikes to the entire GC population. Could the authors discuss the feasibility of such a protocol? In contrast, the protocols used previously (eg ref 22) consist of bursts in single GCs, and measure the efficacy of transmission of these inputs. Furthermore, the synchrony observed in PC population (Fig 9, 11) may thus be a direct result of this massively coincident GC inputs. Additionally ref 45, cited on line 284, does not suggest such coincident GC input as a source of synchronization, but ephaptic coupling via extracellular currents. Is there an alternative reference for this synchronized GC activation?
4. In continuation with the previous point, the burst protocol used here leads to depressed PC responses for longer trains, due to the cooperative facilitation of MLI-mediated inhibition and depression of GC-PC excitation. This is in contrast to experimental data in ref 22. Could the authors fully explain this discrepancy (in light of the additional difference in protocols and synaptic STP profiles)?
5. In Fig 6, temporal profile of PC response is evaluated with 10 Hz stimulations that are lower than most ranges used elsewhere in the paper. Could the authors discuss this choice, especially given that the synaptic dynamics can switch from facilitating to depressing at low to high frequencies? Further, if the aim is to explore reliability of PC spike timings, some of the more direct analyses in Fig S4 may be more relevant than mean ISIs currently included in Fig 6, which only convey the firing rate rather than temporal aspects?
6. The authors suggest that STP enhances reliability of PC spike timings (in Fig 6, S3-4) which may seem intuitively at odds with decreased PC synchronization observed with STP (fig 10). Could the authors discuss the difference in protocols, eg input frequency (10 Hz vs 50-300Hz) used to quantify these response features?

## **Minor points:**

- Could the authors clarify (perhaps in Methods) what turning off STP means, especially as they change U to different levels in Fig 3, in the absence of STP. Specifically, does it mean that the synaptic resource (R) and release (u) are held fixed (at 1 and U respectively), rather than following the dynamics specified? Then changing U would effectively scale the excitatory conductance. Furthermore, could the authors clarify whether STP at other synapses was also removed, or only at GC-PC synapses?
- It would be helpful if justification for the number of neurons of each cell class, and the number of activated cells could be discussed in methods, given that the anatomical expansion ratios are different?
- Line 168-170, and Discussion line 411-413: mentions multiplicative scaling of I-O curves with MLI-dependent inhibition but it actually looks like offset (additive change) in fig 2c . This is also quantified as no gain change in –STP +/- MLI in Fig 2D.
- Line 148: Introduces concept of high dimensional representations at GCs. However these are mostly postulated, and not experimentally verified/established. It would be helpful to cite the main theoretical papers that formulated this hypothesis. Eg: Marr 1969, Albus 1971, Cayco-Gajic and Silver 2019.
- Fig 4&5: Phase shifts – It is unclear how to interpret the relatively modest changes in phase shifts with input frequency, with different synaptic parameters. Example, Could variability (across simulations) or effect size comparision be added?
- Line 64: Possibly wrong reference inserted, should be ref 9 instead of ref 10?
- Figure 1D: Notes 3 example PC traces as well as FR curves. However, it is unclear there are 3 highly overlapping curves. If all cells have nearly identical curves in the absence of inhibition/STP, can a single example/average curve be plotted instead? Or the legend could clarify there are 3 overlapping curves?
- Fig 3B: Legend says PC firing but y-axis label seems to indicate it is net excitatory conductance?
- Figure 5c: For each small panel, indicate what is the frequency range (0 to 30 from top to down, or 30 to 0?) Is it the same as Fig 4?
